# 2dGBH: Two-dimensional group Benjamini–Hochberg procedure for false discovery rate control in two-way multiple testing of genomic data

**DOI:** 10.1093/bioinformatics/btae035

**Published:** 2024-01-19

**Authors:** Lu Yang, Pei Wang, Jun Chen

**Affiliations:** Division of Computational Biology, Department of Quantitative Health Sciences, Mayo Clinic, Rochester, MN 55905, United States; Center for Individualized Medicine, Mayo Clinic, Rochester, MN 55905, United States; Department of Statistics, Miami University, Oxford, OH 45056, United States; Division of Computational Biology, Department of Quantitative Health Sciences, Mayo Clinic, Rochester, MN 55905, United States; Center for Individualized Medicine, Mayo Clinic, Rochester, MN 55905, United States

## Abstract

**Motivation:**

Emerging omics technologies have introduced a two-way grouping structure in multiple testing, as seen in single-cell omics data, where the features can be grouped by either genes or cell types. Traditional multiple testing methods have limited ability to exploit such two-way grouping structure, leading to potential power loss.

**Results:**

We propose a new 2D Group Benjamini–Hochberg (2dGBH) procedure to harness the two-way grouping structure in omics data, extending the traditional one-way adaptive GBH procedure. Using both simulated and real datasets, we show that 2dGBH effectively controls the false discovery rate across biologically relevant settings, and it is more powerful than the BH or *q*-value procedure and more robust than the one-way adaptive GBH procedure.

**Availability and implementation:**

2dGBH is available as an R package at: https://github.com/chloelulu/tdGBH. The analysis code and data are available at: https://github.com/chloelulu/tdGBH-paper.

## 1 Introduction

In clinical omics data analysis, one frequent statistical task is to identify the omics features associated with a disease outcome (Mallick *et al.* 2021). The identified omics features can provide mechanistic insights into the underlying biological and disease processes, and be potentially used as biomarkers for disease prevention, diagnosis, and treatment. Such omics-wide association testing involves large-scale multiple testing and multiple testing procedures such as false discovery rate (FDR) control (Benjamini and Hochberg 1995) or family-wise error rate (FWER) control (Holm 1979) are routinely applied to control for false positives. With the development of new omics technologies, omics studies have become increasingly deeper and broader, producing a new two-way grouping structure for multiple testing. For example, in single-cell omics studies, individual cells are clustered into cell subsets (Kiselev *et al.* 2019), and omics-wide testing is performed for each cell subset. Thus, the individual *P*-values can be grouped by either cell subsets or genes, creating a two-way grouping structure. Similarly, in multi-omics studies of the human microbiome, one routine task is to perform pairwise association testing between microbial features and metabolomic features (Noecker *et al.* 2016, Kim *et al.* 2020). These association *P*-values can be grouped by either microbial features or metabolomic features, leading to another two-way grouping structure (i.e. each microbial [metabolomic] group consists of association *P*-values from the given microbial [metabolomic] feature to all metabolomic [microbial] features). In the single-cell example, the differential signals can be mainly distributed in specific cell subsets or specific genes or both. Similarly, in the multi-omics example, the differential signals can mostly be attributed to specific microbial features or specific metabolomic features or both. Such nonuniform distribution patterns can be potentially leveraged to improve the power of signal detection.

Although the two-way grouping structure has richer structure information, in practice, ordinary multiple testing procedures are applied without taking into full account of the two-way grouping structure. When controlling the FDR, i.e. the expected proportion of false rejections among all rejections, is desired, both global FDR control and stratified FDR control procedures have been applied. In global FDR control, all the *P*-values are pooled, and one-time FDR control is performed. The Benjamini–Hochberg (BH) (Benjamini and Hochberg 1995) procedure and Storey's *q*-value procedure (ST) (Storey 2002) are two most commonly used FDR control methods. The BH procedure is a step-up procedure, which orders the *P*-values from small to large and rejects the largest number of k hypotheses such that the kth *P*-value $P_{\left(k\right)}<k/m\times \alpha$, where m is the total number of tests and α is the target FDR level. Storey's *q*-value approach further considers the proportion of null hypotheses $\pi_{0}$, and finds the largest k such that the $P_{\left(k\right)}<k/m\pi_{0}\times \alpha$. When the signal density is high, the ST procedure is more powerful than the BH procedure. Stratified FDR control, on the other hand, conducts FDR control separately for each stratum (e.g. each cell type or gene) rather than pooling all the *P*-values for global FDR control (Sun *et al.* 2006). This approach allows for a more nuanced correction, as it takes into account the different signal densities within each stratum. Both stratified Benjamini–Hochberg (stratBH) procedure, and stratified Storey's *q*-value (stratST) procedure have been performed for stratified FDR control.

Besides the stratified FDR control procedures, other dedicated group-adaptive FDR control procedures have been developed to increase the power of multiple testing when group structure exists (Hu *et al.* 2010, Sankaran and Holmes 2014, Scott *et al.* 2015, Liu *et al.* 2016, Huang *et al.* 2020, Ignatiadis *et al.* 2016, Boca and Leek 2018, Lei and Fithian 2018, Nandi *et al.* 2021, Sarkar and Nandi 2021, Zhang and Chen 2022). The adaptive group Benjamini–Hochberg procedure (AdaptiveGBH) (Hu *et al.* 2010) is one of the most used procedures for this purpose. AdaptiveGBH implements an adaptive rule to reject the null hypotheses based on the signal density estimate of the group. For features from groups with higher signal density, a larger *P*-value cutoff is imposed to make reject decision. Although the number of false discoveries for those signal-rich groups will be increased by relaxing the rejection criterion, it can be compensated by tightening the rejection criterion for groups with lower signal density. The overall result is an increased detection power while maintaining the FDR level. The procedure is equivalent to performing Benjamini–Hochberg FDR correction at level $q\pi_{0}^{i}$ within each group, where q is the target FDR level and $\pi_{0}^{i}$ is the proportion of null hypotheses within the group i. To estimate the null proportion $\pi_{0}^{i}$, several methods exist including the Least Slope method (Benjamini and Hochberg 2000), the Two Step Test method (Benjamini *et al.* 2006), or Storey tail proportion of *P*-values method (Storey *et al.* 2004). We refer the readers to a recent review on this subject (Kang 2020).

Although stratBH, stratST and AdaptiveGBH can be applied to the two-way grouping structure, they are only capable of using one-way grouping structure. In practice, the user has to decide which grouping structure to use in these procedures. However, the distribution of the signals is usually unknown before the analysis. Signals can be enriched in either direction. Cherry-picking the grouping structure to be used can lead to increased type I error, especially when the signal is sparse. Furthermore, it is unclear whether stratBH and statST can truly control the global FDR in all settings since controlling FDR within each group does not necessarily infer the global FDR control when the detection power is low.

In this study, we propose a 2D Group Benjamini–Hochberg (2dGBH) procedure, an FDR control procedure designed to exploit the two-way grouping structure in omics data. 2dGBH is an extension of the AdaptiveGBH procedure for one-way grouping structure. By extensive evaluation on both simulated and experimental datasets, we show that 2dGBH can effectively control the FDR and adaptive to the underlying signal enrichment pattern. It is overall more robust than AdaptiveGBH and is more powerful than the traditional BH and ST procedure.

## 2 Materials and methods

### 2.1 2dGBH procedure

The 2dGBH procedure is designed to be adaptive to the underlying signal structure and aims to be robust and powerful when the signals are enriched in either or both directions. Suppose we have the *P*-values $P_{ij}\;(i=1,\ldots ,n;j=1,\;\cdots ,\;m)$, where n is the number of features in the first dimension and m is the number of features in the second dimension. With some abuse of terminology, we use the term “outcome” to indicate features in the second dimension. The 2dGBH method consists of the following steps (Supplementary Fig. S1):

1. Estimate the overall proportion of null hypotheses ${(\hat{\pi }}_{0})$ using one of the estimators: Least Slope method (Benjamini and Hochberg 2000) (*lsl*), Two Step Test method (Benjamini *et al.* 2006) (*tst*), or Storey tail proportion of *P*-values method (Storey *et al.* 2004) (*storey*). *storey* is the default method in 2dGBH.
2. Calculate the group-specific proportions of null hypotheses for outcomes $({\hat{\pi }}_{0}^{i,1},\;i=1,\ldots ,n\;$and features $\left({\hat{\pi }}_{0}^{j,2},j=1,\ldots ,m\right)$ using *lsl*, *tst*, or s*torey* method as in the AdaptiveGBH (Hu *et al.* 2010). *storey* is the default method in 2dGBH.
3. Apply a shrinkage factor (S) to both ${\hat{\pi }}_{0}^{i,\;1}$ and ${\hat{\pi }}_{0}^{j,\;2}$ to reduce the estimation variability due to a small number of *P*-values in the group and increase the robustness (better type I error control) of the method. The final estimate is the weighted average of the global and group-specific estimate of the null hypothesis proportions, with the weight determined by the shrinkage factor:
  $${\tilde{\pi }}_{0}^{i,1}=\left(1-S\right)\times {\hat{\pi }}_{0}^{i,1}+S\times {\hat{\pi }}_{0},$$
  $${\tilde{\pi }}_{0}^{j,2}=\left(1-S\right)\times {\hat{\pi }}_{0}^{j,2}+S\times {\hat{\pi }}_{0}.$$
  The default shrinkage factor is 0.1, which increases the robustness of method without significantly affecting its power.
4. Weight *P*-values based on the informativeness of the respective groups. We propose to use the weight
  $$W^{ij}=\frac{1-{\hat{\pi }}_{0}^{ij}}{{\hat{\pi }}_{0}^{ij}},\;$$
  where
  $${\hat{\pi }}_{0}^{ij}={\left({\tilde{\pi }}_{0}^{i,1}\right)}^{1-R}\times {\left({\tilde{\pi }}_{0}^{j,2}\right)}^{R}\text{ and }R=\frac{{\hat{\sigma }}_{{\tilde{\pi }}_{0}}^{\left(2\right)}/\sqrt{m}}{{\hat{\sigma }}_{{\tilde{\pi }}_{0}}^{\left(1\right)}/\sqrt{n}+{\hat{\sigma }}_{{\tilde{\pi }}_{0}}^{\left(2\right)}/\sqrt{m}}.$$

R is determined by the standard deviation of ${\tilde{\pi }}_{0}^{i,1}$ and ${\tilde{\pi }}_{0}^{j,2}$ (${\hat{\sigma }}_{{\tilde{\pi }}_{0}}^{\left(1\right)}$, ${\hat{\sigma }}_{{\tilde{\pi }}_{0}}^{\left(2\right)})$, and the group size of ${\hat{\pi }}_{0}^{i,1}$ and ${\hat{\pi }}_{0}^{j,2\;}$(m and n, respectively). The greater the standard deviation and the larger the group size, the higher the weight the corresponding grouping direction receives. Such weighting scheme is based on the idea that ${\hat{\sigma }}_{{\tilde{\pi }}_{0}}^{\left(1\right)},\;{\hat{\sigma }}_{{\tilde{\pi }}_{0}}^{\left(2\right)}$ measure the informativeness of the respective direction of grouping and the group size is inversely related to the uncertainty of $\pi_{0}^{i,1}$ and $\pi_{0}^{j,2}$ estimates. If the proportions of nulls are similar across the groups (small standard deviation), the signals are more evenly distributed in these groups and less weights will be given for that grouping direction. When R=0 or 1, the weight is only contributed by one direction of grouping. This can happen when the direction of grouping is not very informative (e.g. ${\tilde{\pi }}_{0}^{i,1}$s are similar across features) or the group size is too small (e.g. small number of outcomes).
5. Calculate the weighted *P*-values $P_{ij}^{w}=P_{ij}/W_{ij}\times \left(1-{\hat{\pi }}_{0}^{w}\right),$ where the updated overall proportion of null hypotheses ${\hat{\pi }}_{0}^{w}=\frac{1}{mn}\sum {\hat{\pi }}_{0}^{ij}$.
6. Apply the classic BH method to the weighted *P*-values.

### 2.2 Simulation setup

**Figure 1.**
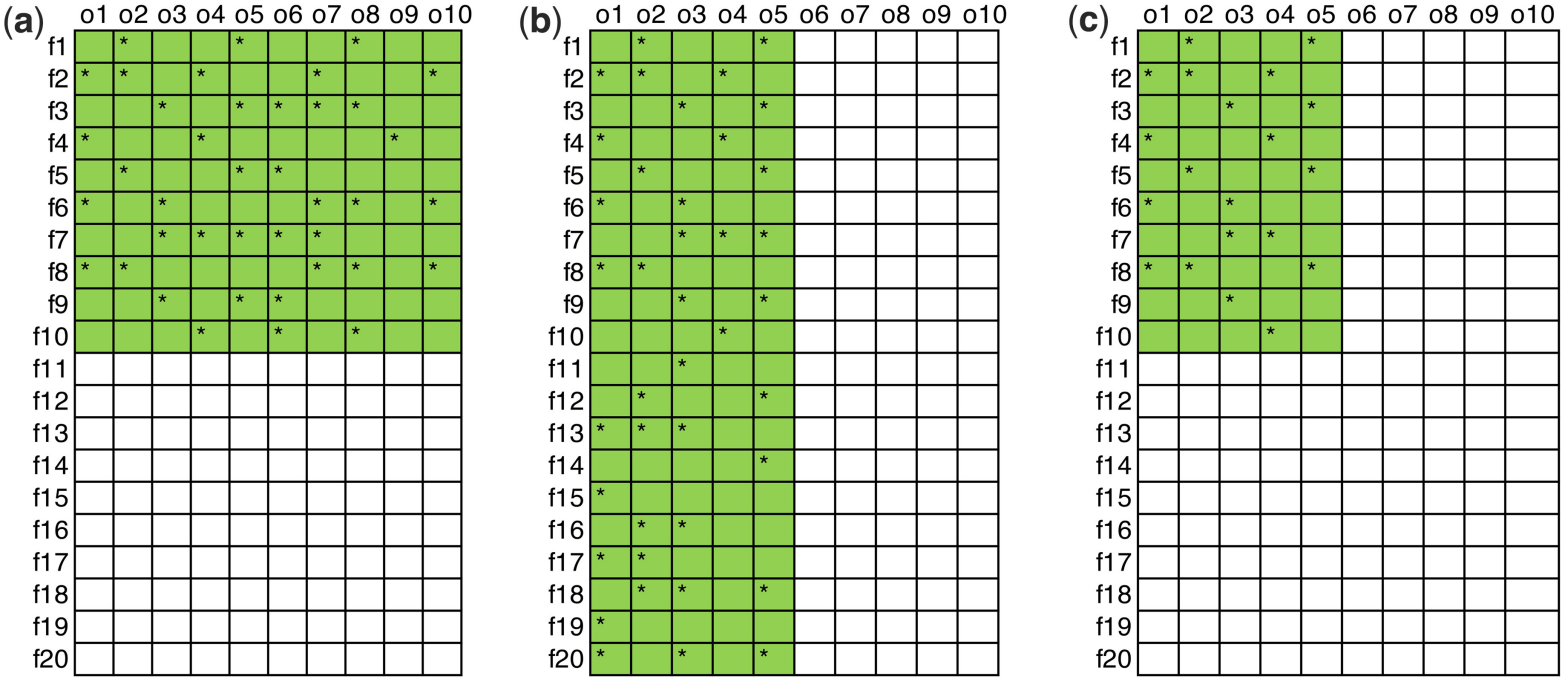
The three signal enrichment patterns investigated in this study. Rows and columns represent features and outcomes. Star(*) indicates the signals (nonnull hypothesis). (a) Signal enriched by feature ($n=20,\;m=\;10,\;\theta =0.2,\;p^{i}=1,\;{\;p}^{j}=0.5$). (b) Signal enriched by outcome (*n* = 20, *m* = 10, $\theta =0.2,\;p^{i}=0.5,\;p^{j}=1$). (c) Signal enriched by both feature and outcome (*n* = 20, *m* = 10, $\theta =0.1,\;p^{i}=0.5,\;{\;p}^{j}=0.5$).

We perform comprehensive simulations to study the performance of the proposed method under different signal enrichment patterns (as described in Fig. 1). We simulate n features (e.g. genes) and m outcomes (e.g. cell types) with an overall signal density (proportion of nonnulls) of θ. Denote the proportions of signal-associated outcomes and features as $p^{i}$ and $p^{j}$.

We start with simulating z-scores $z_{ij}$, which we convert into *P*-values $P_{ij}$ before applying the proposed method. We first investigate the case where $z_{ij}$s are independent and later we will study the correlated cases. We first generate i.i.d. $z_{ij}\;∼\;N\left(0,\;1\right),\;i=1,\ldots ,\;n,\;j=1,\;\ldots ,\;m,$ where $N\left(0,\;1\right)\;$is the distribution of the z-score under the null. Next, based on the overall signal density θ, we simulate i.i.d. $z_{k}\;∼\;N\left(\mu ,\;\sigma^{2}\right),\;k=1,\;\ldots ,\;mn\theta ,$ where $N\left(\mu ,\;\sigma^{2}\right)$ is the distribution under the alternative and $\mu ,\;\sigma^{2}$ control the signal strength and variability. Once we obtain $z_{k}$s, we create different signal enrichment patterns by replacing a subset of $z_{ij}$s with $z_{k}$s (Fig. 1). Specifically, we investigate three scenarios:

1. Signals are enriched in a subset of features (${0<p}^{i}{<1,\;p}^{j}=1$), where we randomly distribute $z_{k}$s in randomly selected ${np}^{i}$ features.
2. Signals are enriched in a subset of outcomes$\;(p^{i}=1,\;{0<p}^{j}<1$), where we randomly distribute $z_{k}$s in randomly selected ${mp}^{j}$ outcomes.
3. Signals are enriched in a subset of outcomes and features (${0<p}^{i}<1,\;{0<p}^{j}<1$), where we randomly distribute $z_{k}$s in randomly selected ${np}^{i}$ outcomes and ${mp}^{j}$ features.

Finally, $z_{ij}$s are converted into *P*-values $P_{ij}$s using the one-sided formula $1-{\Phi (z}_{ij})$, where Φ(.) is the cumulative distribution function of the standard normal. We also study the case, where $z_{ij}$s are correlated. We specifically investigate the block and AR(1) correlation structures. In both cases, we first simulate i.i.d. $\epsilon_{n\times 1}\;∼\;\mathrm{MVN}(0_{n\times 1},\;\Sigma_{n\times n})$ for each outcome, where MVN is a multivariate normal distribution. For block correlation structure, we let $\Sigma_{ii}={1,\;\Sigma }_{ij}=0.7$ if i,j from the same block, and $\Sigma_{ij}=0$ otherwise. For AR(1) correlation structure, we let $\Sigma_{ij}=0.7^{\left|i-j\right|}$. Next, we add the effect $\mu_{ij}$ ($\mu_{ij}=2$, and 0 for alternative and null hypothesis, respectively) similarly as in the independent case. For both the independent and correlated cases, the following parameter settings are investigated:

1. The impact of signal density when signals are associated with a subset of outcomes $(n=1000,\;m=20,\;p^{i}=1,\;p^{j}=0.2,\;\theta \in (0.01,\;0.02,\;0.05,\;0.1)).$
2. The impact of signal density when signals are associated with a subset of features $(n=1000,\;m=20,\;p^{i}=0.2,\;p^{j}=1,\;\theta \in (0.01,\;0.02,\;0.05,\;0.1)).$
3. The impact of signal density when signals are associated with a subset of features and outcomes $(n=1000,\;m=20,\;p^{i}=0.2,\;p^{j}=0.2,\;\theta \in (0.005,\;0.01,\;0.02,\;0.04)).$

In addition, we study the performance under a larger number of outcomes (m=500), a relevant setting for association between two high-dimensional omics data types.

### 2.3 Competing methods

We compare our method to classic FDR control methods, including the Benjamini–Hochberg (BH) Procedure and Storey's *q*-value (ST), and FDR control methods that utilize group structure information, including the Adaptive Group Benjamini–Hochberg Procedure (AdaptiveGBH), stratified Benjamini–Hochberg Procedure (stratBH), stratified Storey's *q*-value (stratST). These group-adaptive methods can only accommodate 1D grouping structure. We include methods using either grouping direction (stratBH_o, stratST_o, and AdaptiveGBH_o for outcome-wise grouping [“_o” represents “outcome”], and stratBH_g, stratST_g, and AdaptiveGBH_g for feature-wise grouping [“_g” represents “genes/features”]). For the evaluation, we utilized the following software packages with default parameter setting: p.adjust function (in R package stats v4.1.2), qvalue function (in R package qvalue v2.26.0). For AdaptiveGBH, we used Adaptive.GBH function with method = ‘storey’ [R package structSSI v1.2.0 (Sankaran and Holmes 2014)].

### 2.4 Performance evaluation

The performance evaluation is based on the FDR control and true positive rate (TPR) with a target FDR level of 5%. The results are averaged over 1000 simulation runs for the global null setting and 100 simulation runs for other settings.

### 2.5 Experimental datasets

We use three experimental datasets to evaluate the performance of 2dGBH. These datasets consist of two microbiome datasets and one single-cell RNA-Seq dataset. The first dataset, “Combo” (Wu *et al.* 2011, Hoffmann *et al.* 2013), is a microbiome dataset studying the relationship between nutrient intake and bacterial genus abundance. Any genus with a prevalence <10% in the samples was removed from analysis, resulting in 37 genera and 214 nutrients for 98 samples, and 7918 tests in total. The second dataset, “Adenoma” (Kim *et al.* 2020), is a microbiome data studying the association between bacterial genus abundance and metabolic sub-pathway abundance. Genera with <10% prevalence in the samples were excluded. The final analysis includes 77 genera and 92 metabolic pathways for 241 samples, and 7084 tests in total. The third dataset, “Autism” (Velmeshev *et al.* 2019), is a single cell RNA Seq (scRNA-Seq) dataset used to find the differential genes between autism subjects and controls across 17 cell types. For each cell type, gene expression was summed across cells per gene per sample, resulting in pseudo-bulk gene expression data for each cell type. Genes expressed in <95% of the cells for each cell type were excluded in the analysis, yielding 3541–16371 (median 7905/mean 9303) genes in the studied cell types, and 158157 tests in total. For both microbiome datasets, we used ZicoSeq (Yang and Chen 2022) to perform association testing. For the scRNA-Seq dataset, we applied GMPR normalization (Chen *et al.* 2018) and performed a Wilcox rank sum test [as suggested by (Li *et al.* 2022)] to detect differential genes for each cell types. The *P*-values obtained are used as the input to 2dGBH and the competing methods.

## 3 Results

### 3.1 Simulation studies

#### 3.1.1 Performance under the global null setting

**Figure 2.**
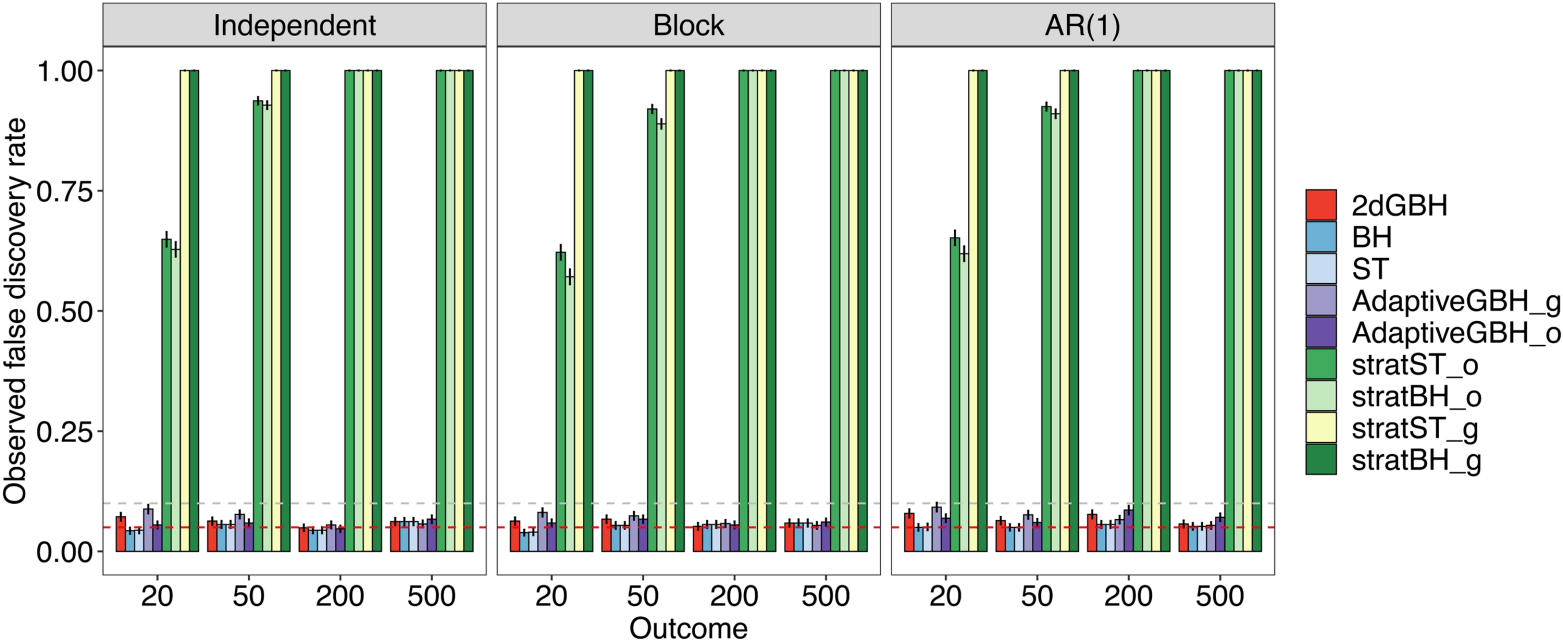
Performance of 2dGBH and its competing methods under the global null setting. Performance is assessed by the observed false discovery rate (FDR) level, calculated as the percentage of the 1000 simulation runs making any discoveries. 5% target FDR level is used. The two dashed lines represent 5% and 10% FDR level, respectively. BH: Benjamini–Hochberg Procedure, ST: Storey's *q*-value procedure, AdaptiveGBH: Adaptive Group BH Procedure, stratBH: stratified BH Procedure, stratST: Stratified ST procedure. The suffix “_o” represents outcome-wise grouping, and “_g” represents gene/feature-wise grouping.

We first study the performance of the methods under the global null setting, where there are no true signals, and all hypotheses are from the null. Different correlation structures [Independent, Block and AR(1)] are investigated. A robust method should control the FDR closer to the target level. In the global null setting, FDR is equivalent to the probability of making any positive findings by definition. Thus, a robust method is expected to have approximately 5% chance of finding any significant associations if 5% target FDR level is used. In Fig. 2, we show that both 2dGBH and the traditional FDR control methods (BH and ST) control the FDR near the target level across settings. In contrast, stratBH and stratST have substantial FDR inflation. When the number of outcomes is large, the chance of false positive findings is close to 100%, meaning that these stratified methods will always retrieve some significant associations even if the dataset has no signal. Thus, applying stratified FDR control should be cautious when the number of strata is large. On the other hand, both versions of AdaptiveGBH display comparable FDR levels as 2dGBH.

#### 3.1.2 Performance under the independent setting when there are association signals

**Figure 3.**
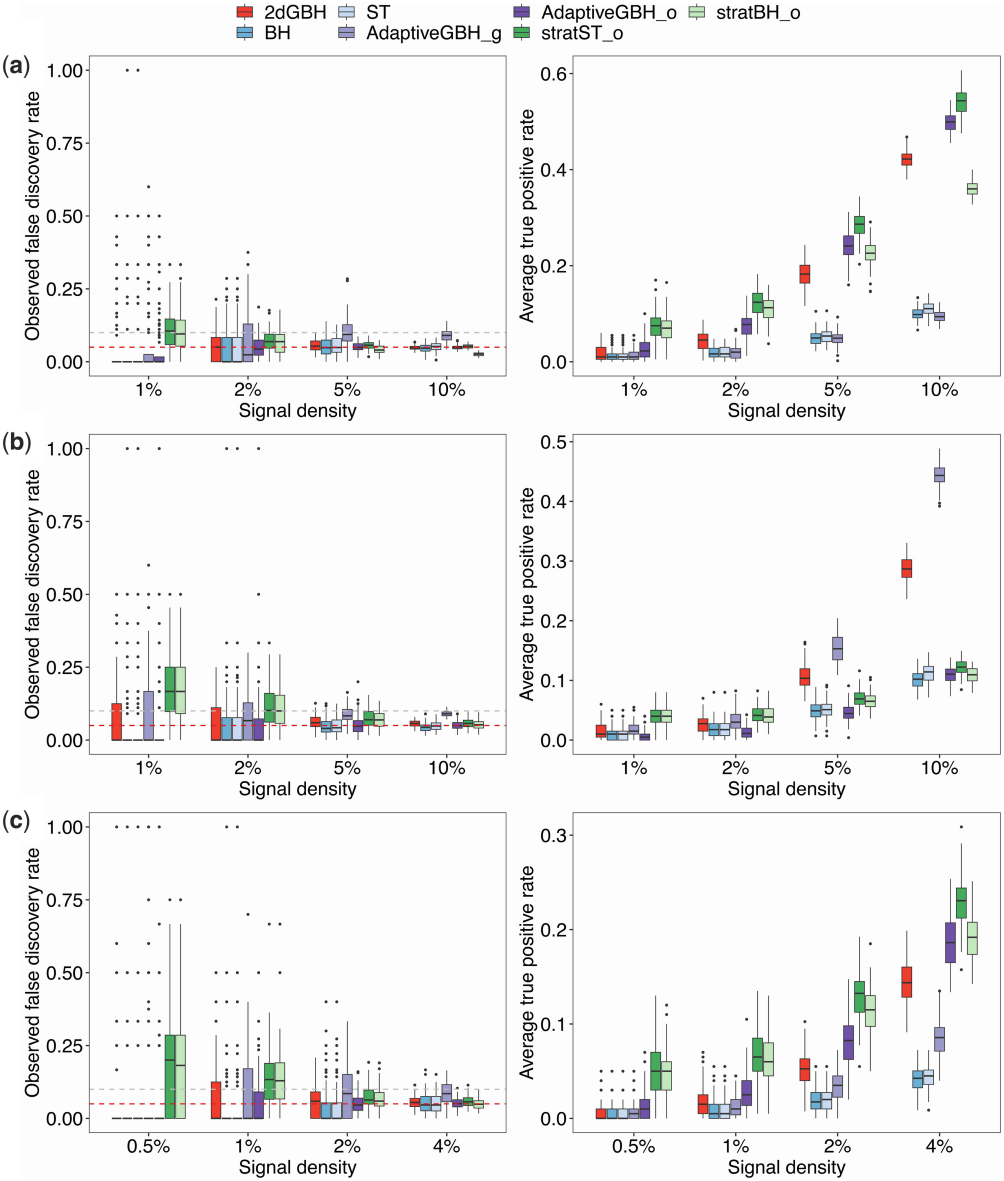
Performance of 2dGBH and its competing methods under the independent setting with 20 outcomes. 5% target FDR is used. (a) Signals are only associated with a subset of outcomes $\left(n=1000,\;{m=20,\;p}^{i}=0.2,\;p^{j}=1,\;\theta \in (0.01,\;0.02,\;0.05,\;0.1)\right)$. (b) Signals are only associated with a subset of features $(n=1000,m=20,p^{i}=1,\;p^{j}=0.2,\;\theta \in (0.01,\;0.02,\;0.05,\;0.1)).\;$(c) Signals are associated with a subset of features and outcomes $(n=1000,\;m=20,\;p^{i}=0.2,\;p^{j}=0.2,\;\theta \in (0.005,\;0.01,\;0.02,\;0.04)).\;$Performance is assessed by the observed false discovery rate (FDR) level and average true positive rate (TPR). The two dashed lines represent 5% and 10% FDR level, respectively. BH: Benjamini–Hochberg Procedure, ST: Storey’s *q*-value procedure, AdaptiveGBH: Adaptive Group BH Procedure, stratBH: stratified BH Procedure, stratST: Stratified ST procedure. The suffix “_o” represents outcome-wise grouping, and “_g” represents gene/feature-wise grouping.

We next evaluate the performance of 2dGBH and their competitors under independent setting with signals present and enriched in various patterns. The number of outcomes simulated (m=20) reflects the typical number of cell types in single-cell RNA-Seq (scRNA-Seq) data types. Since the performance of stratBH_g and stratST_g far worse than strateBH_o and stratST_o, we did not include them in comparison. When signals are enriched by outcome (Fig. 3a), 2dGBH, as well as AdaptiveGBH_o, BH, and ST, effectively control the FDR around 5%. The stratBH_o and stratST_o methods both show inflated FDR levels. The inflation increases with decreasing signal density. Thus, application of stratified BH and ST is not advised when the signal content is low. AdaptiveGBH_g displays slightly higher FDR inflation than 2dGBH, probably due to a large number of groups used. In terms of power for those FDR-controlled methods, 2dGBH demonstrates significantly higher power than BH and ST, and is slightly less powerful than AdaptiveGBH_o. The difference from AdaptiveGBH_o decreases with the increasing signal density. The power of AdaptiveGBH_g is similar to BH/ST since the group structure is not informative. Note that when the signal density is high, stratified ST has the highest power with well controlled FDR.

When signals are enriched by feature (Fig. 3b), the FDR control performance is similar to the case with enrichment by outcome (Fig. 3a). AdaptiveGBH_g has the highest power, though slight FDR inflation is noted. AdaptiveGBH_o is substantially less powerful than 2dGBH due to the use of uninformative group structure. When signals are enriched by both feature and outcome, 2dGBH, AdaptiveGBH_o, BH, and ST again control the FDR around the target level (Fig. 3c), while AdaptiveGBH_g, stratBH_o and stratST_o show more FDR inflation than 2dGBH. 2dGBH is more powerful than BH, ST, and AdaptiveGBH_g, but is less powerful than AdaptiveGBH_o. Stratified ST is the most powerful with controlled FDR when the signal density is high (4%).

Next, we simulate 500 outcomes, mirroring the application of pairwise association testing between two high-dimensional datasets, such as the association between microbial taxa abundance and metabolomic abundance. Although some slight differences have been noted (e.g. AdaptiveGBH_g has less inflation), the overall trend remains the same (Supplementary Fig. S2).

Taken together, although 2dGBH is not the most powerful in each setting, its performance is the most robust and the power is always higher than BH/ST. In comparison, AdaptiveGBH_g and AdaptiveGBH_o could be significantly less powerful when the group structure is not informative. Stratified BH/ST work only when the signal density is not very low. In practice, we do not have prior knowledge about the signal enrichment pattern, application of 2dGBH thus is a reasonable choice.

#### 3.1.3 Performance under the correlated settings when there are association signals

In practice, *P*-values can be correlated due to shared influences or inherent dependencies in the data. For instance, block correlations could arise from common factors affecting a set of variables, while AR(1) correlations could manifest in spatial and temporal sampling where outcomes depend on preceding results. Recognizing these correlations is essential for robust data analysis. To further study the robustness of 2dGBH to different correlation structures, we examine its performance under two correlation structures: block correlation (Supplementary Figs S3 and S4) and AR(1) correlation (Supplementary Figs S5 and S6). In general, 2dGBH's performance is as robust as in the independent structure, maintaining FDR control around the target level across all settings, regardless of signal enrichment pattern, and its power is comparable to or surpassing that of competing methods. In contrast, other group-adaptive methods are less robust. For example, stratBH and stratST have severe FDR inflation in low-signal setting. AdaptiveGBH_g has noticeable FDR inflation when the number of outcomes is small (m=20).

#### 3.1.4 Comparison to more alternatives

We perform additional numerical experiments, comparing different ways to combine the marginal weights, and comparing to more existing methods. The settings are mainly the same as those used in the main comparison (20 outcomes, 1000 features, independent setting).

We first examine the effects of the shrinkage factor on the model performance. As shown in Supplementary Fig. S7, although an increase of the shrinkage factor value improves the FDR control when the number of outcomes is small, it reduces the statistical power significantly when the signal density is high. We thus use 0.1 as the default shrinkage factor value, achieving more robustness without affecting the power much.

We also compare 2dGBH to two simpler options to combine the marginal weights: the geometric mean (2dGBH-geo) and arithmetic mean (2dGBH-ari) (Supplementary Fig. S8). Both approaches assign equal weights to the two dimensions. Results indicate that our current weighting method outperforms these naïve ones, especially when the signal density is high. Interestingly, when the signals are clustered by both outcomes and features, 2dGBH is still more powerful than 2dGBH-geo and 2dGBH-ari. Thus, differential weighting based on the informativeness of the respective dimension and the dimension sizes can improve the statistical power.

One reviewer brought to our attention a previously developed two-way GBH method by Nandi *et al.* (2021). In their implementation, two versions were provided: one that places equal emphasis on row and column weights (NSC_1) and the other that accounts for the difference in numbers of rows and columns (NSC_2), both of which do not consider the informativeness of the respective dimension. Results suggest that 2dGBH is significantly more powerful than NCS_1 and NSC_2 in most settings (Supplementary Fig. S9). The tight FDR control of NCS_1 and NSC_2 is at a great expense of power.

Finally, we compare 2dGBH to regression-based covariate-adaptive FDR control methods, including science-wise false discovery rate (swfdr) (Boca and Leek 2018), FDRreg method with empirical (FDRregE) and theoretical (FDRregT) null hypothesis (Scott *et al.* 2015), and covariate adaptive multiple testing procedure (CAMT) (Zhang and Chen 2022). For those methods, we use two categorical covariates to represent the outcome and feature groups and let the prior null probability depend on these two covariates. For swfdr, we used lm_qvalue function (in R package swfdr v1.20.0). For FDRreg, we applied FDRreg function with nulltype = “theoretical” and nulltype = “emprical” along with other default settings (in R package v0.2–1). For CAMT, we employed camt.fdr function with alg.type = “EM” and control.method = “knockoff+” (in R package CAMT v1.1). Results show that regression-based methods fail to maintain the FDR at the expected level (Supplementary Fig. S10). The inability of these regression-based methods to control FDR is attributed to potential overfitting when they involve a large number of parameters and there are not sufficient data to estimate the parameters reliably. For the example with 20 outcomes (regardless of the number of features), only 20 data points are available to estimate the parameter for each feature, which is not enough for these regression methods to work.

### 3.2 Application to real datasets

To demonstrate the practical application of the 2dGBH method, we next apply 2dGBH and its competing methods to three publicly available datasets. Since the ground truth is unknown for the three real datasets, we aim to assess whether the discovery pattern on the real datasets reflects what we have observed in the simulation study.

**Figure 4.**
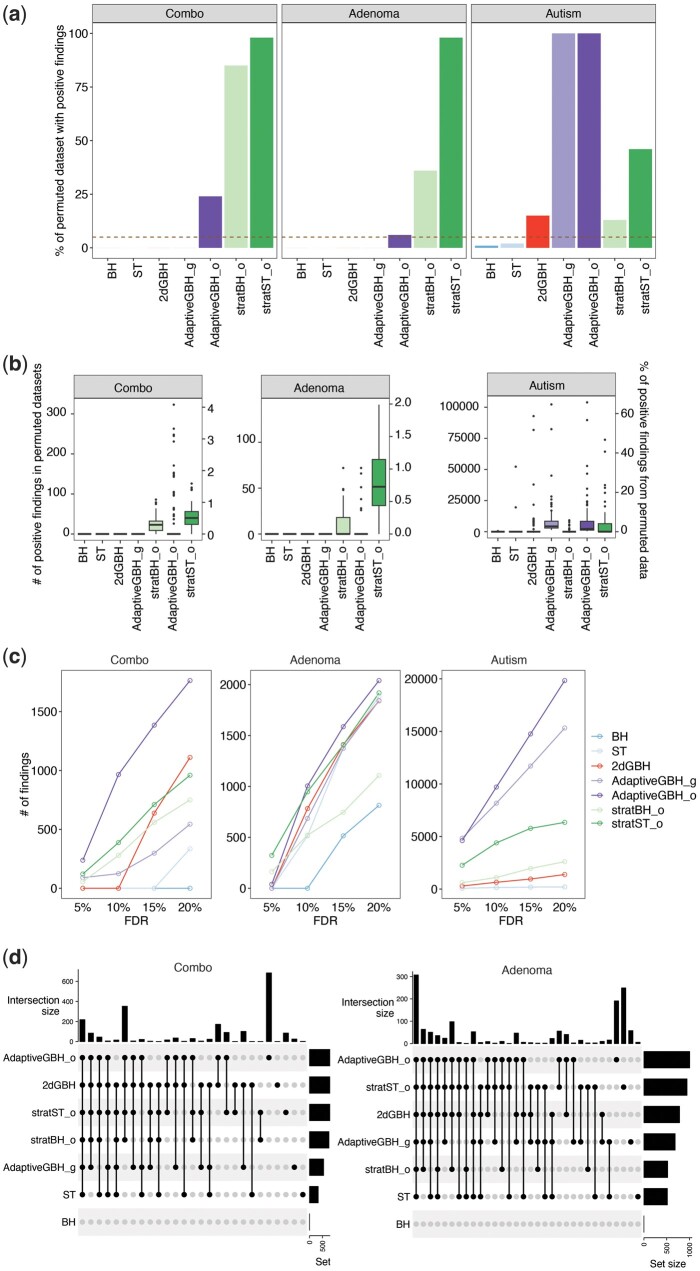
Performance of 2dGBH and its competing methods based on three experimental datasets. (a) Bars showing the observed false discovery rate (FDR) level, calculated as the percentage of the 100 simulation runs making any discoveries. (b) Boxplot showing the numbers of positive findings (left y-axis) and the proportion of positive findings out of all features (right y-axis) based on 100 permuted datasets. (c) Boxplot showing the number of findings at different target FDR levels for the real datasets. (d) Overlaps of significant findings between 2dGBH and its competing methods on the real datasets (“Combo”: 20% target FDR, “Adenoma”: 10% target FDR). Set size means the total number of findings discovered by each method. Intersection size means the number of findings commonly found by the methods indicated by the black dots. BH: Benjamini–Hochberg Procedure, ST: Storey’s *q*-value procedure, AdaptiveGBH: Adaptive Group BH Procedure, stratBH: stratified BH Procedure, stratST: Stratified ST procedure. The suffix “_o” represents outcome-wise grouping, and “_g” represents gene/feature-wise grouping.

We first evaluate the FDR control under the global null by shuffling the sample labels (100 times) to disrupt the differential signals. For the “Combo” dataset, we permute subjects' nutrient intake values; for the “Adenoma” dataset, we permute each subject's metabolic pathway abundance; and for the “Autism” dataset, we shuffle group labels (autism versus control). We then perform association testing and use the raw *P*-values as the input to 2dGBH and its competing methods. Any significant associations after FDR adjustment are considered false positives. Using 5% target FDR level, we expect to see an average of 5% of permuted datasets with positive findings. As anticipated, 2dGBH control FDR close to the target level while stratified BH/ST has severe FDR inflation across datasets, AdaptiveGBH_o and AdaptiveGBH_g have severe FDR inflation for “Combo” (m=37) and “Autism” (m=17 420) dataset, respectively (Fig. 4a and b). The results are consistent with the simulation findings.

Next, we compare the numbers of identified associations at different FDR levels for 2dGBH and its competing methods (Fig. 4c), and study their overlaps based on the original real datasets (Fig. 4d). Consistent with the simulation study, BH and ST tend to find the smallest number of findings, while stratBH and stratST show the highest power (Fig. 4c). For the “Combo” dataset, although 2dGBH could not find any differential taxa at 5%–10% FDR, it has the highest power at the FDR level of 15% or 20% among the methods that control FDR (Fig. 4c). It also has considerable overlaps in findings with other methods (Fig. 4d). For the “Adenoma” dataset, except for AdaptiveGBH_o, stratBH and stratST, no method discovers any differential taxa at 5%. When we increase the target FDR to 10% or higher, 2dGBH generally shows the highest power among those the permuted datasets.

In summary, the detection patterns on the real datasets are generally consistent with the simulation findings. 2dGBH is robust and powerful for real data applications.

## 4 Conclusion

In this study, we present a new approach, 2dGBH, to conduct FDR control when the data exhibits two-way grouping structure. Our comprehensive evaluation across simulated settings and real datasets demonstrates the robustness and power of the 2dGBH. In simulation studies, under both global null, independent and correlated structures, 2dGBH consistently controlled the false discovery rate (FDR) at or around the target level, exhibiting superior performance in comparison to competing methods. Particularly in scenarios of substantial outcome numbers and low signal content, other methods, such as stratified BH/ST, exhibited FDR inflation, risking higher false-positive findings. When applied to real datasets, the performance patterns mirrored our simulation results: 2dGBH exhibited robust FDR control and demonstrated decent power in identifying associations, especially when compared to the traditional methods such as BH and ST. Notably, even though 2dGBH might not always be the most powerful method in all settings, it effectively balances the trade-off between FDR control and power, making it a feasible option for datasets where signal enrichment patterns are unclear.

## Supplementary Material

btae035_Supplementary_Data
