# Supplementary material for: 2dGBH: Two-dimensional group Benjamini–Hochberg procedure for false discovery rate control in two-way multiple testing of genomic data
Source: Bioinformatics. 2024 Jan 19;40(2):btae035. doi: 10.1093/bioinformatics/btae035 (PMC10873908; doi:10.1093/bioinformatics/btae035)
Supplement: btae035_Supplementary_Data [file btae035_supplementary_data.docx]

# Supplementary figures for

# 2dGBH: Two-dimensional Group Benjamini-Hochberg Procedure for False Discovery Rate Control in Two-Way Multiple Testing of Genomic Data

Lu Yang^1,2^, Pei Wang^3^ and Jun Chen^1,2*^

^1^Division of Computational Biology, Department of Quantitative Health Sciences, Mayo Clinic, Rochester, MN, 55905, U.S.A.

^2^Center for Individualized Medicine, Mayo Clinic, Rochester, MN, 55905, U.S.A.

^3^Department of Statistics, Miami University, Oxford, OH 45056, U.S.A.

^∗^To whom correspondence should be addressed.


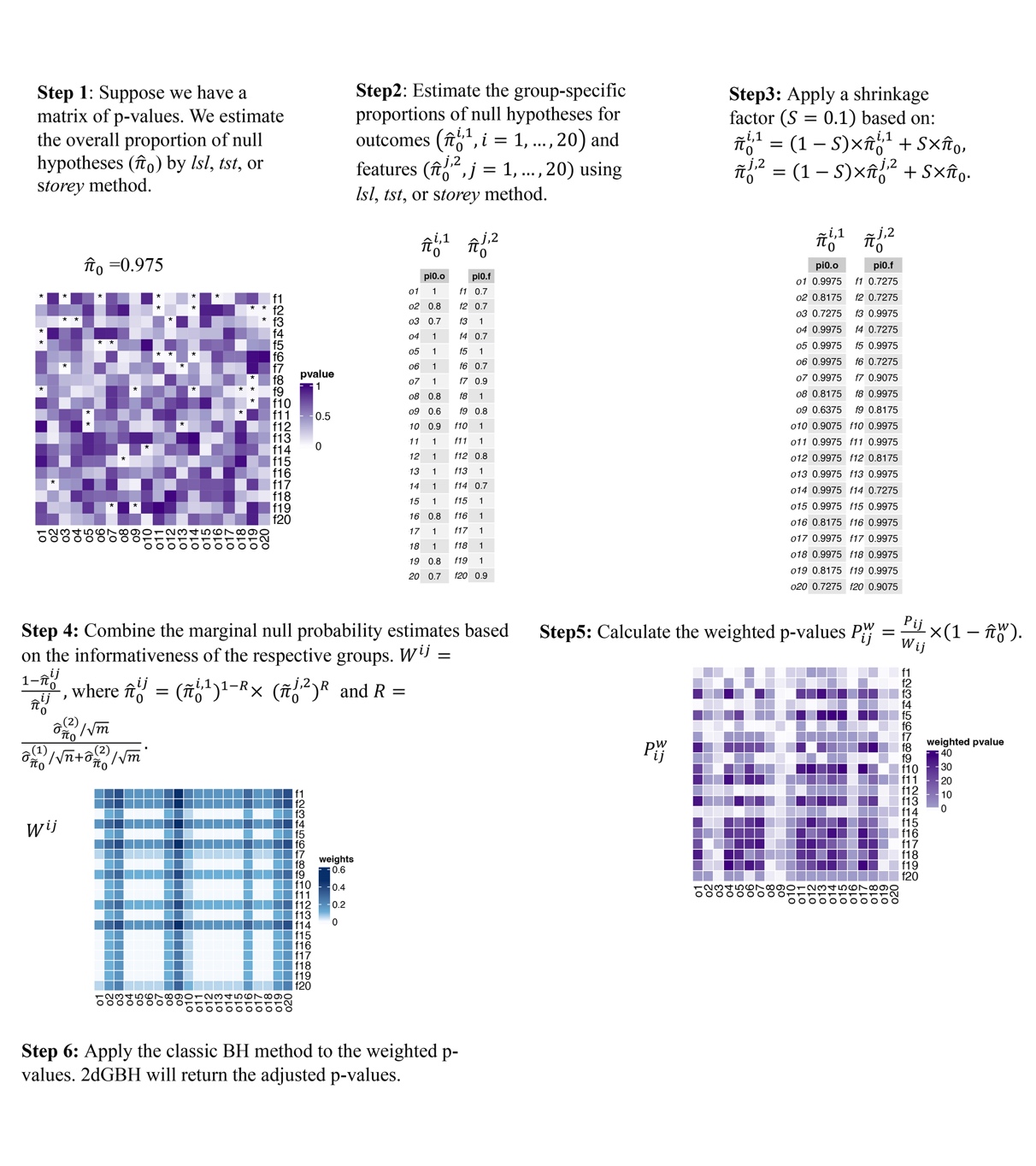


Figure S1. 2dGBH procedures.


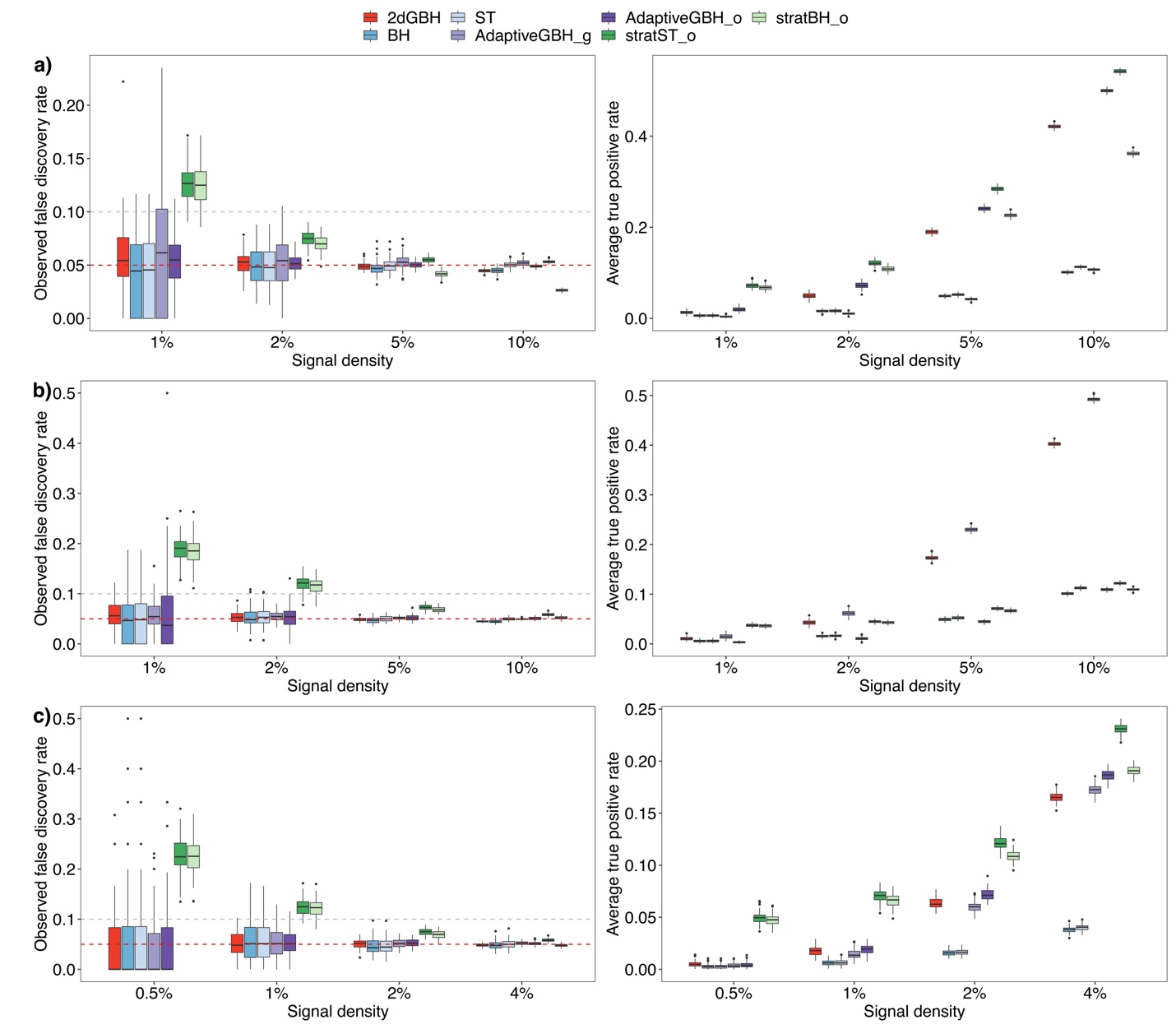


Figure S2. Performance of 2dGBH and its competing methods under the independent setting with 500 outcomes. (A) Signals are only associated with a subset of outcomes $(n=1000,m=500, p^{i}=0.2, p^{j}=1, \theta\in( 0.01, 0.02, 0.05, 0.1))$. (B) signals are only associated with a subset of features $(m=1000,n=500,p^{i}=1, p^{j}=0.2, \theta\in( 0.01, 0.02, 0.05, 0.1)).$ (C) Signals are associated with a subset of features and outcomes $(m=1000,$ $n=1000, p^{i}=0.2, p^{j}=0.2, \theta\in(0.005, 0.01, 0.02, 0.04)).$Performance is assessed by the observed false discovery rate (FDR) level and average true positive rate (TPR). The red and gray line represents FDR level at 0.05 and 0.1, respectively. BH: Benjamini-Hochberg Procedure, ST: Storey's q-value procedure, AdaptiveGBH: Adaptive Group BH Procedure, stratBH: stratified BH Procedure, stratST: Stratified ST procedure. The suffix "_o" represents outcome-wise grouping, and "_g" represents gene/feature-wise grouping.


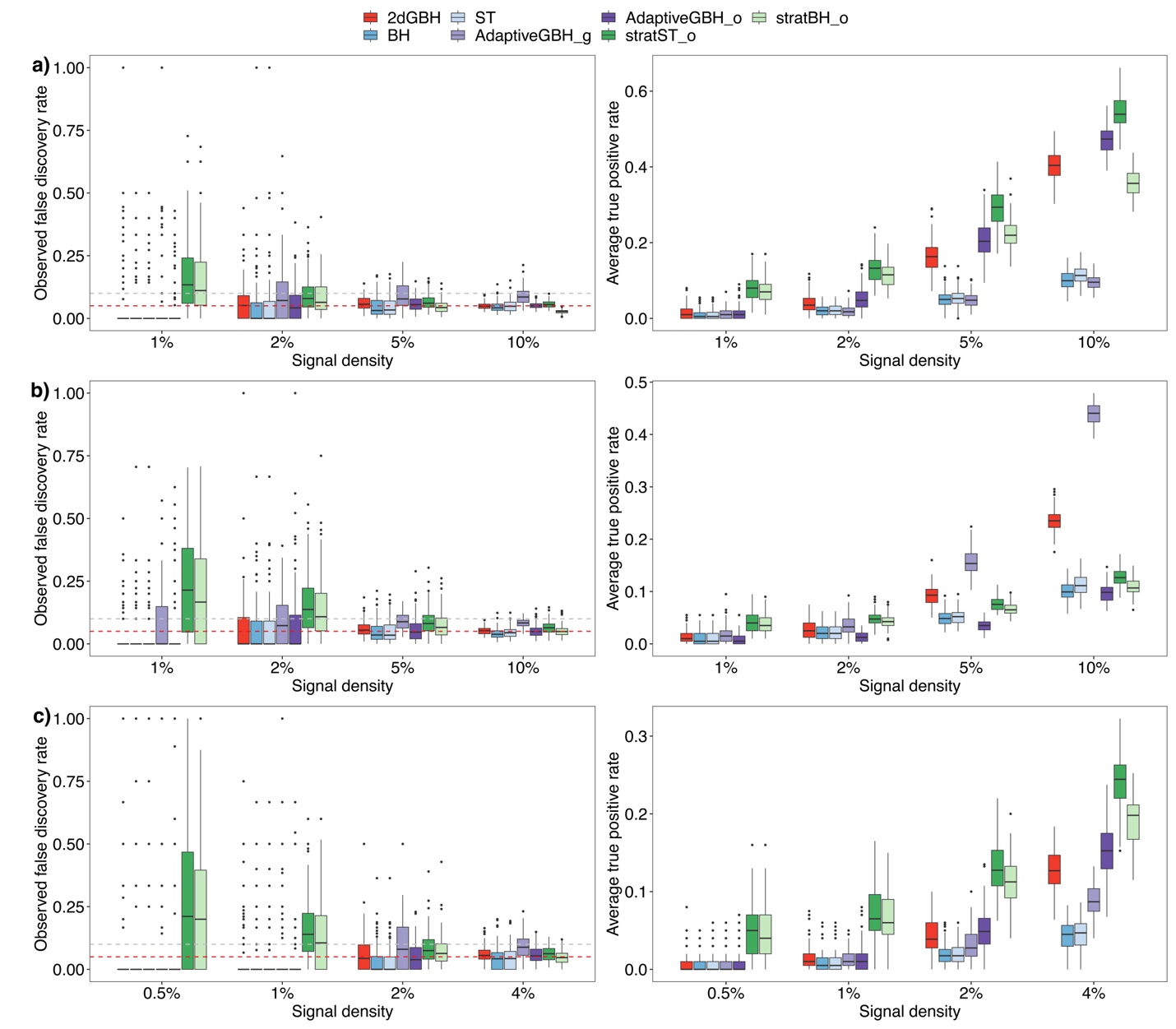
Figure S3. Performance of 2dGBH and its competing methods under the Block correlation structure with 20 outcomes. (a) Signals are only associated with a subset of outcomes $(n=1000,n=20, p^{i}=0.2, p^{j}=1, \theta\in( 0.01, 0.02, 0.05, 0.1))$. (b) Signals are only associated with a subset of features $(n=1000,n=20,p^{i}=1, p^{j}=0.2, \theta\in( 0.01, 0.02, 0.05, 0.1)).$ (c) Signals are associated with a subset of features and outcomes $(n=1000,$ $n=20, p^{i}=0.2, p^{j}=0.2, \theta\in(0.005, 0.01, 0.02, 0.04)).$Performance is assessed by the observed false discovery rate (FDR) level and average true positive rate (TPR). The red and gray line represent 5% and 10% FDR level, respectively. BH: Benjamini-Hochberg Procedure, ST: Storey's q-value procedure, AdaptiveGBH: Adaptive Group BH Procedure, stratBH: stratified BH Procedure, stratST: Stratified ST procedure. The suffix "_o" represents outcome-wise grouping, and "_g" represents gene/feature-wise grouping.


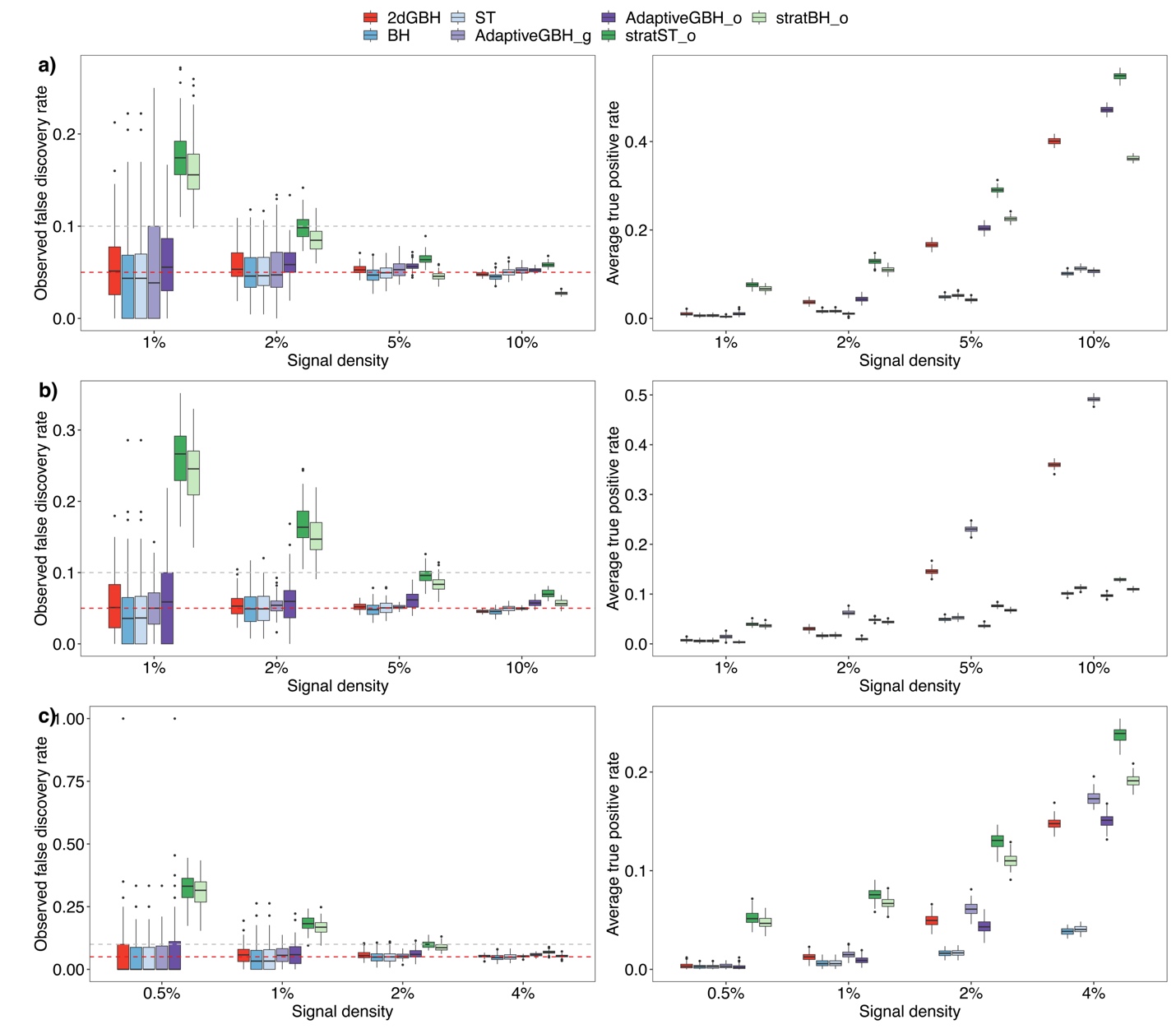
Figure S4. Performance of 2dGBH and its competing methods under the Block correlation structure with 500 outcomes. (a) Signals are only associated with a subset of outcomes $(n=1000,m=500, p^{i}=0.2, p^{j}=1, \theta\in( 0.01, 0.02, 0.05, 0.1))$. (b) Signals are only associated with a subset of features $(n=1000,m=500,p^{i}=1, p^{j}=0.2, \theta\in( 0.01, 0.02, 0.05, 0.1)).$ (c) Signals are associated with a subset of features and outcomes $(n=1000,$ $m=500, p^{i}=0.2, p^{j}=0.2, \theta\in(0.005, 0.01, 0.02, 0.04)).$Performance is assessed by the observed false discovery rate (FDR) level and average true positive rate (TPR). The red and gray line represent 5% and 10% FDR level, respectively. BH: Benjamini-Hochberg Procedure, ST: Storey's q-value procedure, AdaptiveGBH: Adaptive Group BH Procedure, stratBH: stratified BH Procedure, stratST: Stratified ST procedure. The suffix "_o" represents outcome-wise grouping, and "_g" represents gene/feature-wise grouping.


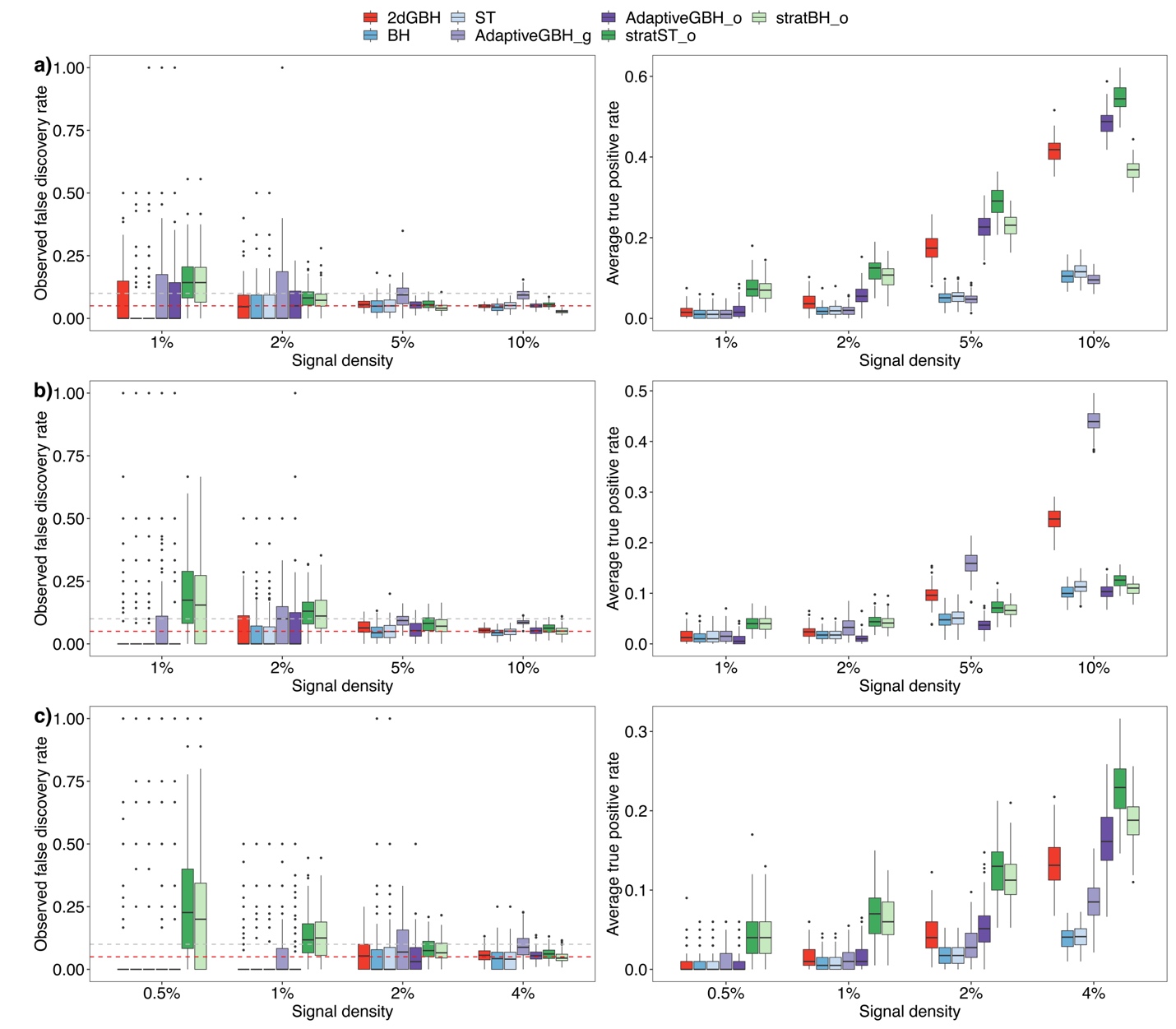
Figure S5. Performance of 2dGBH and its competing methods under the AR(1) correlation structure with 20 outcomes. (a) Signals are only associated with a subset of outcomes $(n=1000,m=20, p^{i}=0.2, p^{j}=1, \theta\in( 0.01, 0.02, 0.05, 0.1))$. (b) Signals are only associated with a subset of features $(n=1000,m=20,p^{i}=1, p^{j}=0.2, \theta\in( 0.01, 0.02, 0.05, 0.1)).$ (c) Signals are associated with a subset of features and outcomes $(n=1000,$ $m=20, p^{i}=0.2, p^{j}=0.2, \theta\in(0.005, 0.01, 0.02, 0.04)).$Performance is assessed by the observed false discovery rate (FDR) level and average true positive rate (TPR). The red and gray line represent 5% and 10% FDR level, respectively. BH: Benjamini-Hochberg Procedure, ST: Storey's q-value procedure, AdaptiveGBH: Adaptive Group BH Procedure, stratBH: stratified BH Procedure, stratST: Stratified ST procedure. The suffix "_o" represents outcome-wise grouping, and "_g" represents gene/feature-wise grouping.


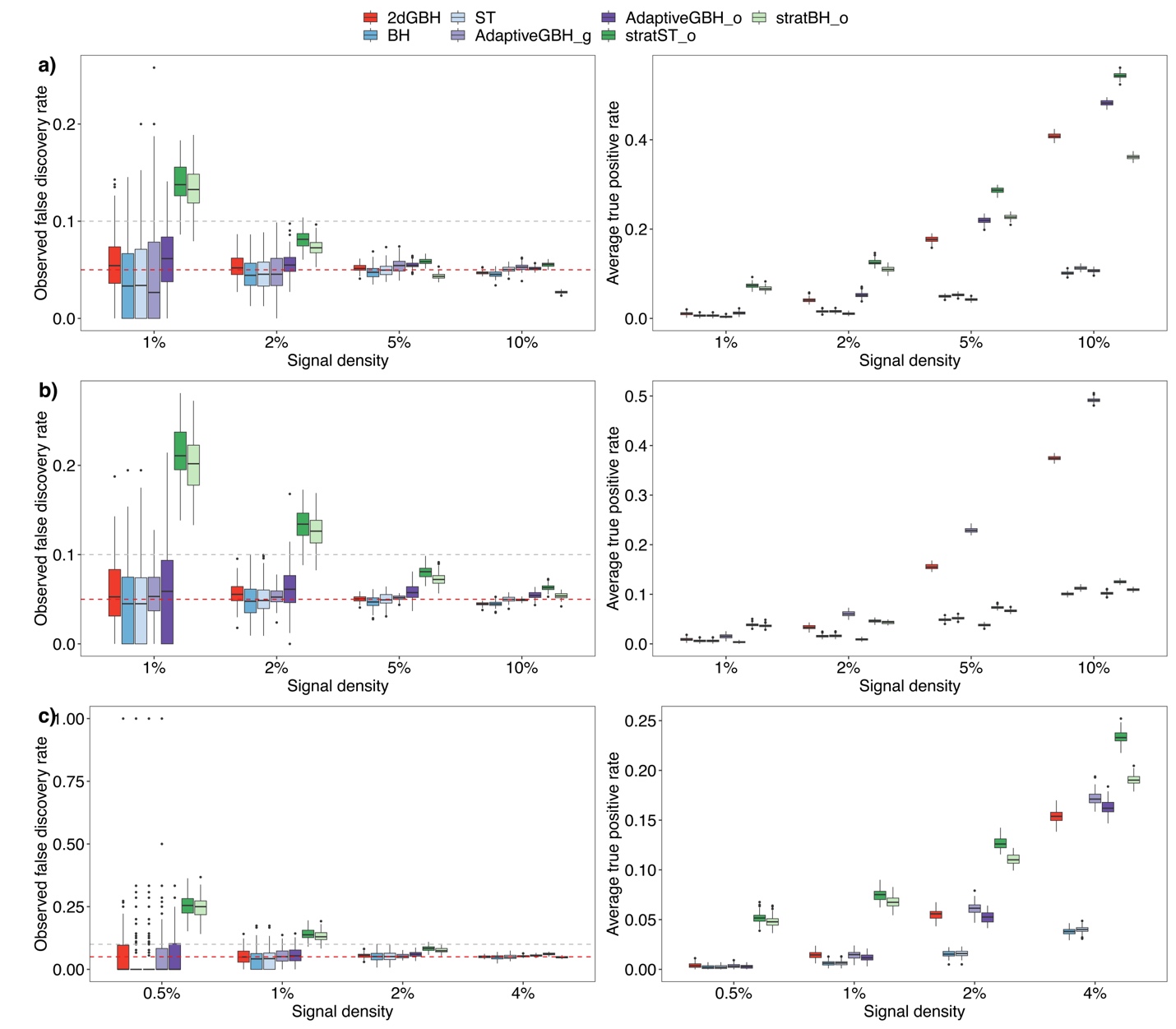
Figure S6. Performance of 2dGBH and its competing methods under the AR(1) correlation structure with 500 outcomes. (a) Signals are only associated with a subset of outcomes $(n=1000,m=500, p^{i}=0.2, p^{j}=1, \theta\in( 0.01, 0.02, 0.05, 0.1))$. (b) Signals are only associated with a subset of features $(n=1000,m=500,p^{i}=1, p^{j}=0.2, \theta\in( 0.01, 0.02, 0.05, 0.1)).$ (c) Signals are associated with a subset of features and outcomes $(n=1000,$ $m=500, p^{i}=0.2, p^{j}=0.2, \theta\in(0.005, 0.01, 0.02, 0.04)).$Performance is assessed by the observed false discovery rate (FDR) level and average true positive rate (TPR). The red and gray line represent 5% and 10% FDR level, respectively. BH: Benjamini-Hochberg Procedure, ST: Storey's q-value procedure, AdaptiveGBH: Adaptive Group BH Procedure, stratBH: stratified BH Procedure, stratST: Stratified ST procedure. The suffix "_o" represents outcome-wise grouping, and "_g" represents gene/feature-wise grouping.


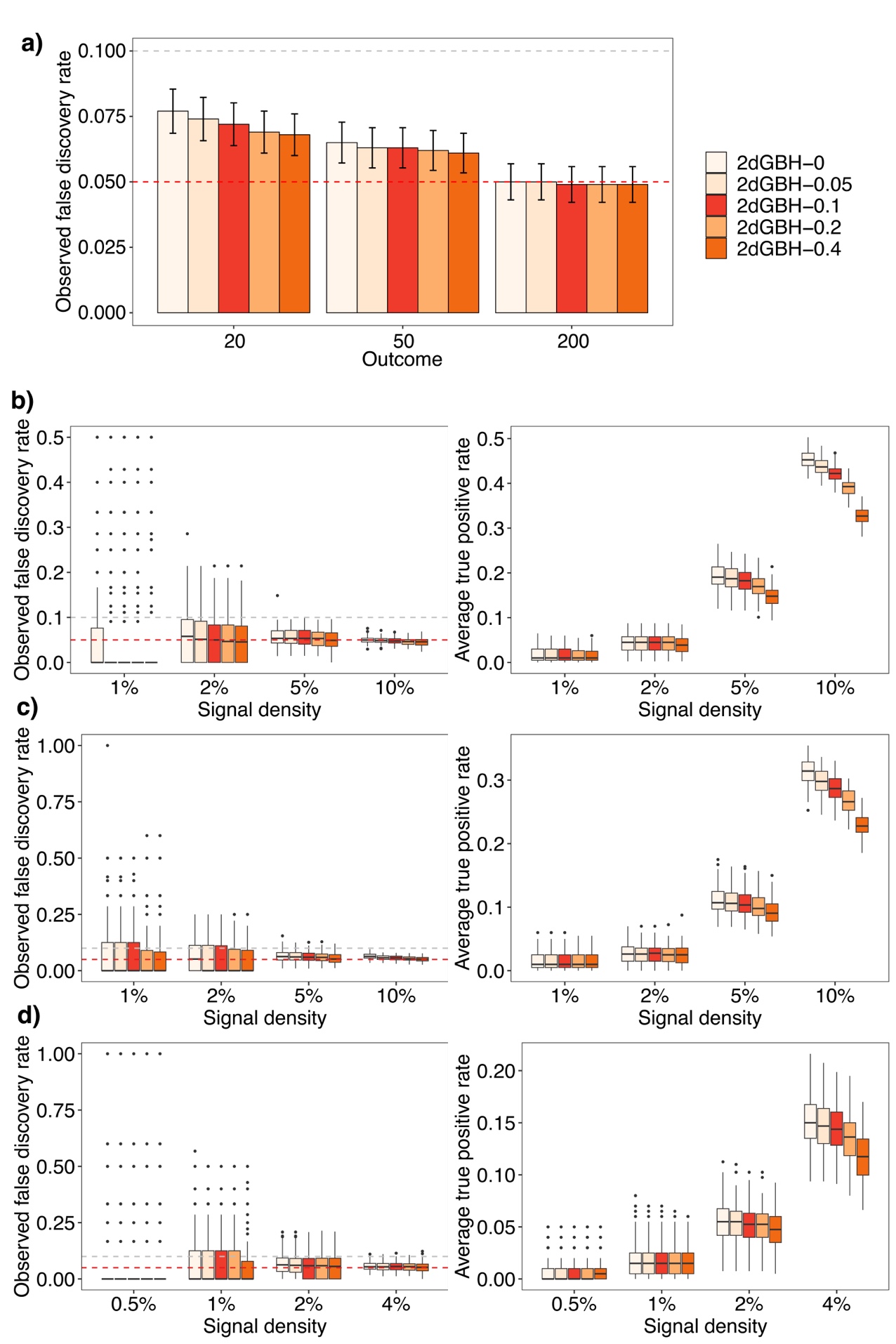


Figure S7. Performance of 2dGBH with different shrinkage factors under the independent setting with 20 outcomes. 5% target FDR is used. (a) Performance of 2dGBH with different shrinkage factors under the global null setting. Performance is assessed by the observed false discovery rate (FDR) level, calculated as the percentage of the 1000 simulation runs making any false discoveries. (b) Signals are only associated with a subset of outcomes $(n=1000, {m=20, p}^{i}=0.2, p^{j}=1, \theta\in( 0.01, 0.02, 0.05, 0.1))$. (c) Signals are only associated with a subset of features $(n=1000,m=20,p^{i}=1, p^{j}=0.2, \theta\in( 0.01, 0.02, 0.05, 0.1)).$ (d) Signals are associated with a subset of features and outcomes $(n=1000,$ $m=20, p^{i}=0.2, p^{j}=0.2, \theta\in(0.005, 0.01, 0.02, 0.04)).$Performance is assessed by the observed false discovery rate (FDR) level and average true positive rate (TPR). 5% target FDR level is used. The red and gray line represent 5% and 10% FDR level, respectively.


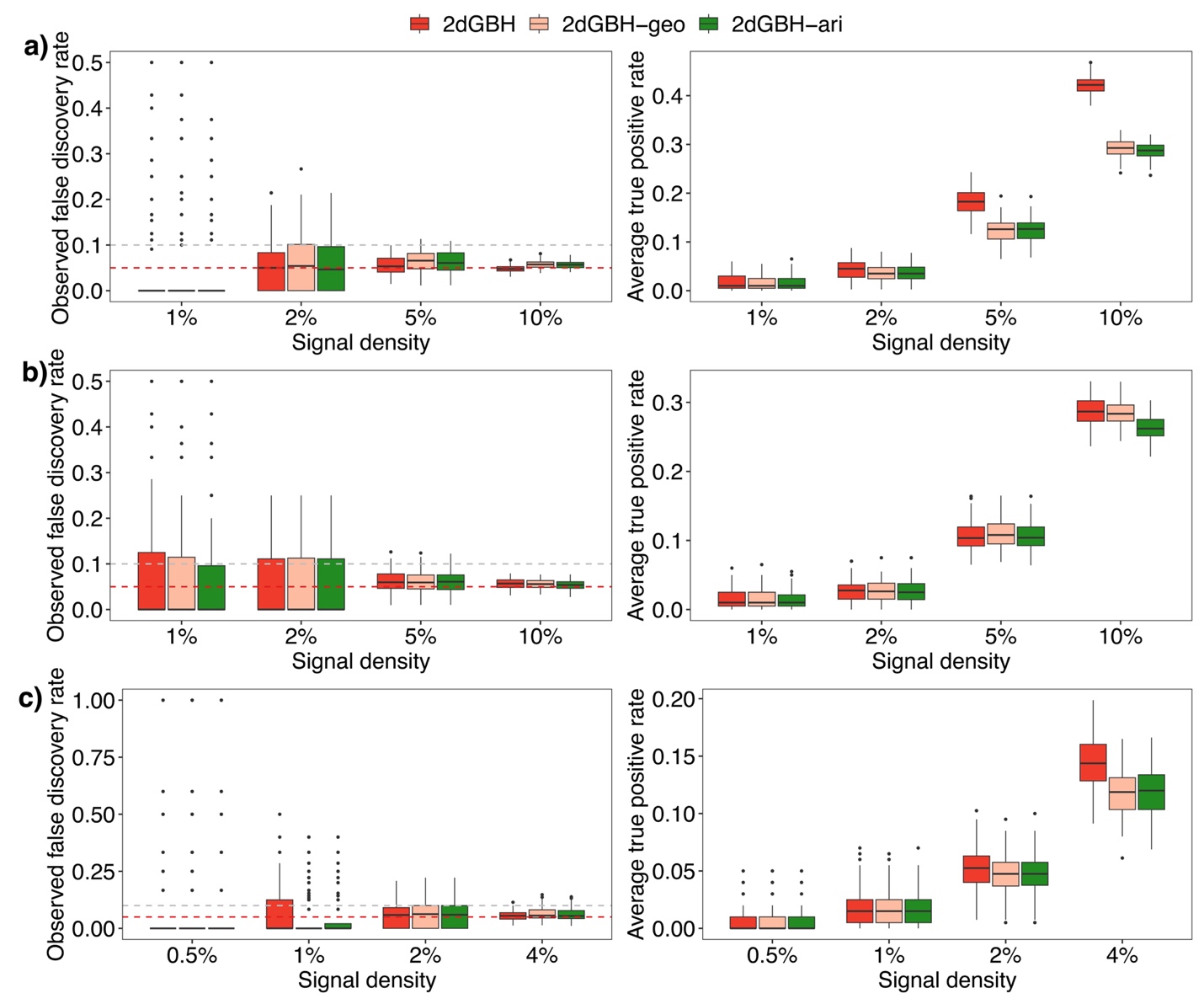
Figure S8. Performance of 2dGBH in comparison with other naïve weighting methods under the independent setting with 20 outcomes. 2dGBH-geo and 2dGBH-ari denote the geometric and arithmetic mean combinations for marginal weights, respectively. 5% target FDR is used. (a) Signals are only associated with a subset of outcomes $(n=1000, {m=20, p}^{i}=0.2, p^{j}=1, \theta\in( 0.01, 0.02, 0.05, 0.1))$. (b) Signals are only associated with a subset of features $(n=1000,m=20,p^{i}=1, p^{j}=0.2, \theta\in( 0.01, 0.02, 0.05, 0.1)).$ (c) Signals are associated with a subset of features and outcomes $(n=1000,$ $m=20, p^{i}=0.2, p^{j}=0.2, \theta\in(0.005, 0.01, 0.02, 0.04)).$Performance is assessed by the observed false discovery rate (FDR) level and average true positive rate (TPR). The red and gray line represent 5% and 10% FDR level, respectively.


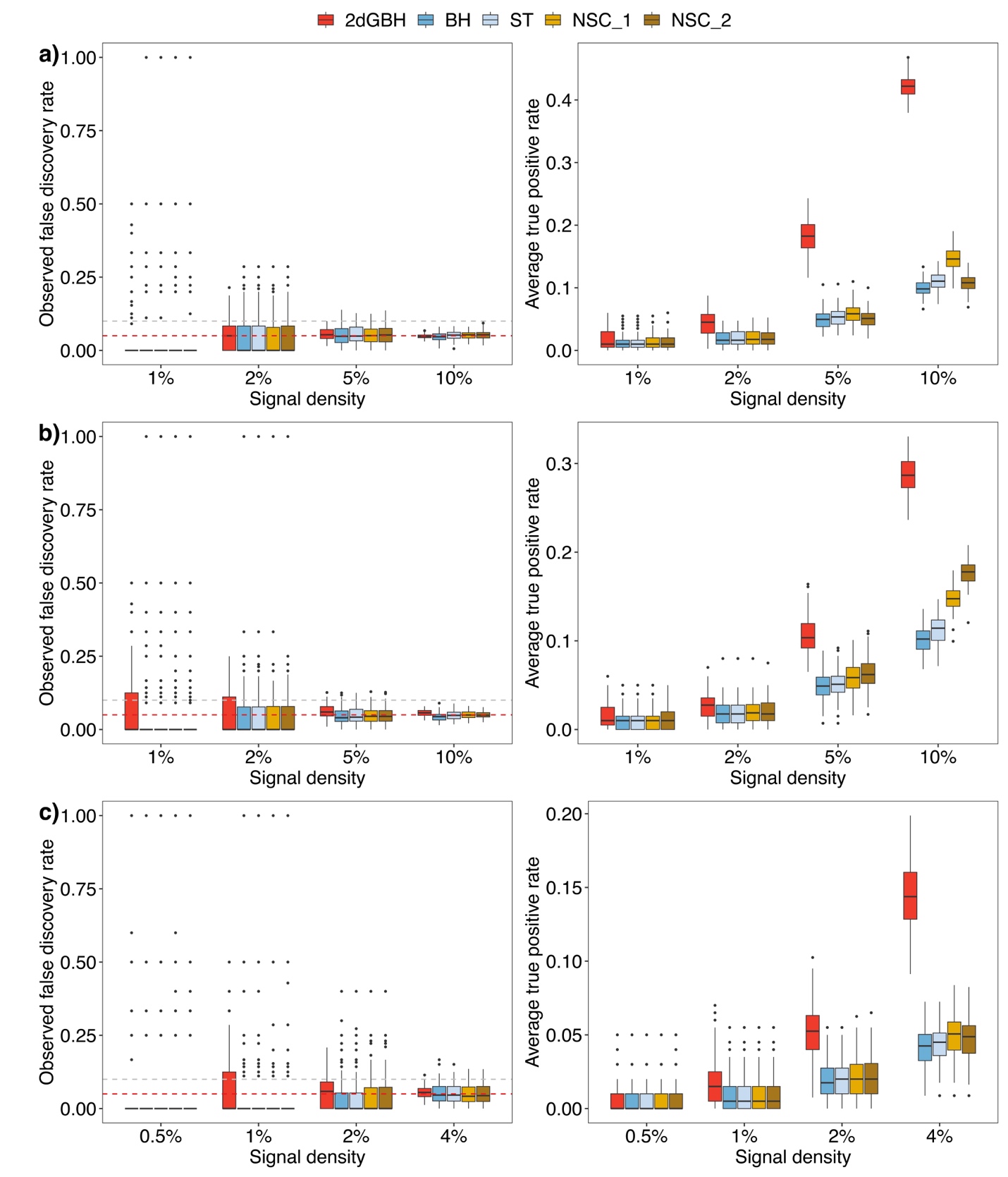


Figure S9. Performance of 2dGBH and previously published two-way GBH methods (NCS_1 and NCS_2) under the independent setting with 20 outcomes. 5% target FDR is used. (a) Signals are only associated with a subset of outcomes $(n=1000, {m=20, p}^{i}=0.2, p^{j}=1, \theta\in( 0.01, 0.02, 0.05, 0.1))$. (b) Signals are only associated with a subset of features $(n=1000,m=20,p^{i}=1, p^{j}=0.2, \theta\in( 0.01, 0.02, 0.05, 0.1)).$ (c) Signals are associated with a subset of features and outcomes $(n=1000,$ $m=20, p^{i}=0.2, p^{j}=0.2, \theta\in(0.005, 0.01, 0.02, 0.04)).$Performance is assessed by the observed false discovery rate (FDR) level and average true positive rate (TPR). The red and gray line represent 5% and 10% FDR level, respectively.


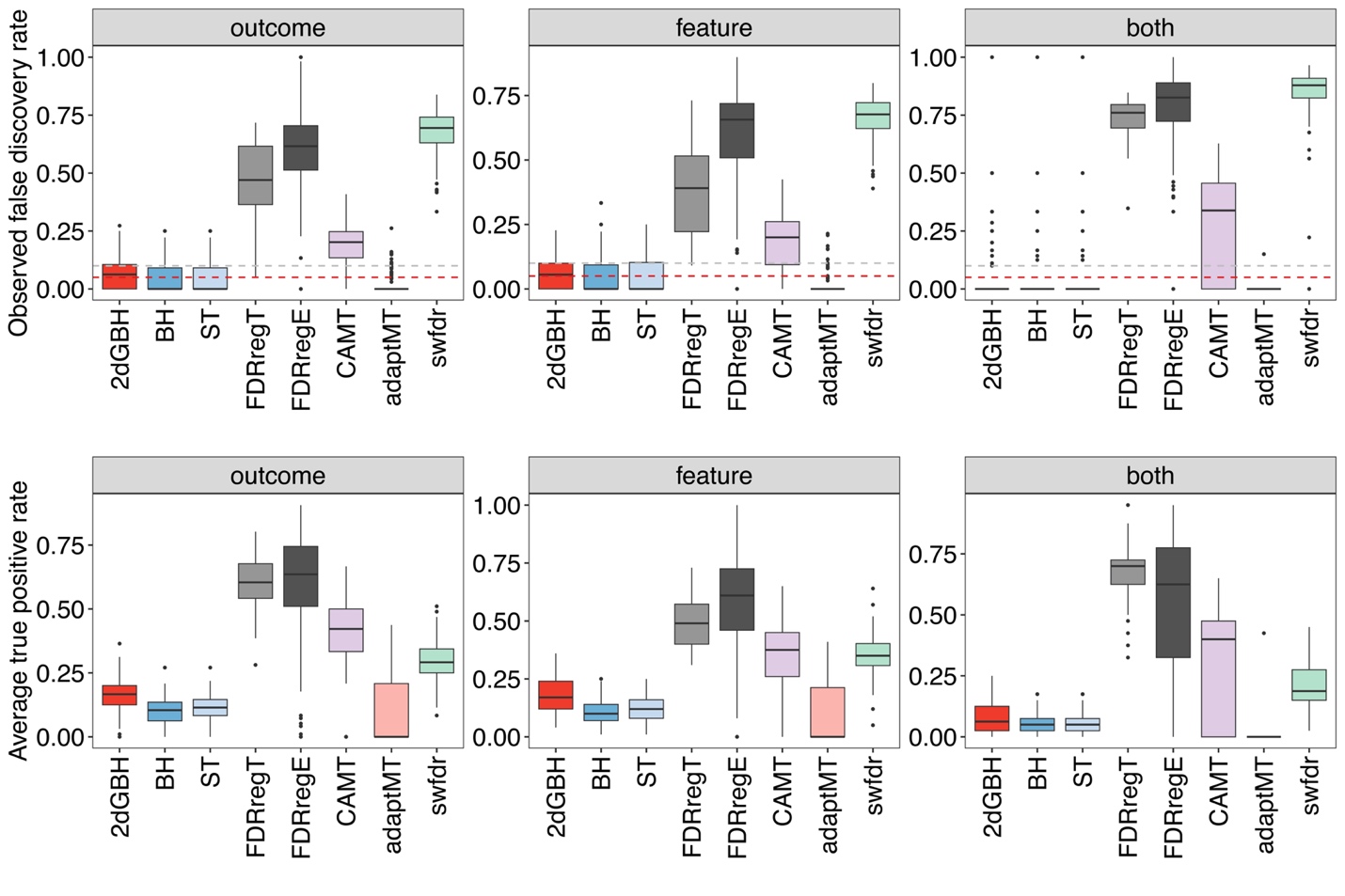


Figure S10. Performance of 2dGBH and regression-based methods under the independent setting with 20 outcomes. 5% target FDR is used. (outcome) Signals are only associated with a subset of outcomes $(n=50, {m=20, p}^{i}=0.2, p^{j}=1, \theta=0.1)$. (feature) Signals are only associated with a subset of features $(n=50,m=20,p^{i}=1, p^{j}=0.2, \theta=0.1).$ (both) Signals are associated with a subset of features and outcomes $(n=1000,$ $m=20, p^{i}=0.2, p^{j}=0.2, \theta= 0.04).$Performance is assessed by the observed false discovery rate (FDR) level and average true positive rate (TPR). The red and gray line represent 5% and 10% FDR level, respectively.
